# Association of X-ray Repair Cross Complementing Group 1 Arg399Gln Polymorphisms with the Risk of Squamous Cell Carcinoma of the Head and Neck: Evidence from an Updated Meta-Analysis

**DOI:** 10.1371/journal.pone.0077898

**Published:** 2013-10-30

**Authors:** Yadong Wang, Xinwei Chu, Xiaojing Meng, Fei Zou

**Affiliations:** 1 Henan Center for Disease Control and Prevention, Zhengzhou, China; 2 Department of Nutrition Health, School of Public Health and Tropic Medicine, Southern Medical University, Guangzhou, China; 3 Department of Occupational Health and Occupational Medicine, School of Public Health and Tropic Medicine, Southern Medical University, Guangzhou, China; Baylor College of Medicine, United States of America

## Abstract

**Background:**

Epidemiologic studies have reported the association of X-ray repair cross-complementary group 1 (*XRCC1*) Arg399Gln polymorphisms with susceptibility to squamous cell carcinoma of the head and neck (HNSCC). However, the results were conflictive rather than conclusive. The purpose of this study was to clarify the association of *XRCC1* Arg399Gln variants with HNSCC risk.

**Methods:**

Systematic searches were performed through the search engines of PubMed, Elsevier, Science Direct, CNKI and Chinese Biomedical Literature Database. Summary odds ratio (OR) with 95% confidence intervals (CI) was computed to estimate the strength association.

**Results:**

Overall, we did not observe any association of *XRCC1* Arg399Gln polymorphisms with HNSCC risk in total population (OR = 0.95, 95% CI: 0.76–1.19 for Gln/Gln vs. Arg/Arg, OR = 1.05, 95% CI: 0.92–1.20 for Arg/Gln vs. Arg/Arg, and OR = 1.03, 95% CI: 0.90–1.18 for Gln/Gln+Arg/Gln vs. Arg/Arg) based on 18 studies including 3917 cases and 4560 controls. In subgroup analyses, we observed an increased risk of *XRCC1* 399 Arg/Gln genotype for HNSCC in Caucasians (OR = 1.20, 95% CI: 1.00–1.44) and Gln/Gln genotype for larynx squamous cell carcinoma (OR = 1.63, 95% CI: 1.10–2.40). We did not observe any association between *XRCC1* Arg399Gln variants and HNSCC risk in additional subgroup analyses.

**Conclusion:**

The results from this present meta-analysis suggest that *XRCC1* Arg399Gln variants may contribute to HNSCC risk among Caucasians and to the risk of larynx squamous cell carcinoma. Further, well-designed studies with larger sample sizes are required to verify our findings.

## Introduction

The global incidence of squamous cell carcinoma of the head and neck (HNSCC) is 540,000 per year, with an annual mortality rate of 271,000. HNSCC is a malignant disease of the upper aerodigestive tract, including oral, pharyngeal and laryngeal regions. HNSCC occurs most commonly in men between the sixth and seventh decade of life. Epidemiologic data have shown that tobacco smoking and alcohol consumptions are the main etiological factors in the carcinogenesis of squamous cell carcinoma of the head and neck [1].

Although a majority of people are exposed to such risk factors, only a small fraction of them develop HNSCC [2]. This indicates that an individual’s susceptibility might play a certain role in HNSCC carcinogenesis. Recently, Increasing evidence has been accumulated to support the hypothesis that common genetic polymorphisms in genes involved in DNA repair capacity may be of importance in determining an individual’s susceptibility to develop HNSCC [3]–[6].

XRCC1 is a major DNA repair gene involved in base excision repair (BER) and single-strand breaks (SSBs) repair. XRCC1 interacts strongly with Poly [ADP-ribose] polymerase 1 (PARP1), which recognizes SSBs, and LIGIII that seals SSBs and BER intermediates [7]. Several single nucleotide polymorphisms (SNP) have been identified in the XRCC1 gene, three of which (Arg194Trp, Arg280His and Arg399Gln) occur within conserved sequences, as described by Shen et al [8]. A polymorphism Arg399Gln (rs25487) leads to amino acid substitutions (exon 10, G-A). This mutation could alter XRCC1 function, diminish repair kinetics, and influence susceptibility to adverse health effect, such as cancer. To date, a number of studies have investigated the association between *XRCC1* Arg399Gln polymorphisms and HNSCC risk [1], [3], [5], [9]–[23]. However, the results from epidemiologic studies have been inconsistent. Moreover, Li et al [5] conducted a meta-analysis on the association of *XRCC1* Arg399Gln polymorphisms with HNSCC risk in 2007 and did not observe any significant association based on 7 published studies. Ever since, several new studies have provided additional data on the association between *XRCC1* Arg399Gln polymorphisms and HNSCC risk. In order to address a more precise estimation of this relationship, a meta-analysis including a total of 18 studies was performed, which may provide the more comprehensive evidence for the association of *XRCC1* Arg399Gln variants with HNSCC risk.

## Materials and Methods

### Literature and Methods

Systematic searches in Pubmed/Medline, Elsevier, Science Direct, Chinese National Knowledge Infrastructure (CNKI) and Chinese Biomedical Literature Database were performed using the following search terms: “head and neck cancer” or “oral cancer” or “larynx cancer” or “pharynx cancer” and “polymorphism” and “*XRCC1*”. Additional studies were identified using the reference lists of the selected papers. The ending date of publication search was March 1, 2013.

The following criteria were used for study selection: (1) The papers should evaluate the association between *XRCC1* Arg399Gln polymorphisms and HNSCC risk; (2) Case-control studies or cohort studies; (3) Sufficient data were used for estimating OR with 95% CI; (4) When more than one article was identified for the same study population, we included the publication containing more information. The exclusion criteria were (1) studies on nasopharyngeal cancer; (2) studies that could not offer the number of cases and controls or other essential information; (3) reviews or studies with overlapping patient populations; (4) studies without histologically confirmed information for HNSCC.

In total, 30 published articles were identified with the association between *XRCC1* Arg399Gln polymorphisms and head and neck cancer risk. We reviewed all papers in accordance with the criteria listed, above; seven studies without histologically confirmed information for HNSCC, three overlapping studies and two reviews were excluded. At last, 18 original articles that reported the association between *XRCC1* Arg399Gln polymorphisms and HNSCC risk were determined to be eligible to enter our study (Figure S1 Flow diagram).

### Data Extraction

Data were carefully extracted and tabulated by two of the authors independently, and then inputted into an electronic database. The following information was subtracted from each study: author’s name, publishing date, country, ethnicity of subjects, source of control, total number of cases and controls and number of every genotype. If the study provided stratum information, the data coming from similar stratum were added up to make full use of the data. Characteristics of selected studies were summarized in Table 1.

**Table 1:** Studies on the association of *XRCC1* Arg399Gln polymorphisms with HNSCC risk included in meta-analysis.

First author	Year	controlsource	Country	Ethnicity	Tumor site	No. ofCase	No. ofControl	*P* value of HWE
Applebaum [9]	2009	Healthy	USA	Unknown	HNSCC	483	547	0.762
Bogela [10]	2011	Hospital	China	Asian	Larynx	58	116	0.542
Csejtei [11]	2009	Healthy	Hungary	Caucasians	HNSCC	108	102	0.985
Demokan [12]	2005	Healthy	Turkey	Unknown	Oral cavity	95	98	0.922
Dos Reis [13]	2013	Healthy	Brazil	Brazilian	Oral cancer	150	150	0.001
Gugatschka [3]	2011	Healthy	Austria	Caucasians	HNSCC	168	463	0.240
Harth [14]	2008	Healthy	Germany	Caucasians	Oral cavity, pharynx,larynx	310	300	0.189
Kietthubthew [15]	2006	Hospital	Thailand	Asian	Oral cavity	106	164	0.724
Kostrzewska-Poczekai [1]	2013	Healthy	Poland	Caucasians	HNSCC	290	158	0.550
Kowalski [16]	2009	Hospital	Poland	Caucasians	HNSCC	92	124	0.253
Krupa [17]	2011	Hospital	Poland	Unknown	Larynx	253	253	0.238
Kumar [18]	2012	Healthy	Indian	Asian	HNSCC	278	278	0.132
Li [5]	2007	Hospital	USA	Non-HispanicWhite	Oral cavity,oro/hypopharynx, larynx	830	854	0.577
Majumder [23]	2007	Health	India	Asian	Oral cavity	309	385	0.254
Olshan [19]	2002	Hospital	Unknow	White andblack	oral cavity, pharynx,larynx	98	161	0.182
Tae [20]	2004	Hospital	Korea	Asian	Oral cavity,oro/hypopharynx, larynx	129	157	0.250
Varzim [21]	2003	Healthy	Portugal	Caucasians	Larynx	88	178	0.759
Yang [22]	2008	Hospital	China	Asian	Larynx	72	72	0.763

HWE: Hardy-Weinberg equilibrium.

### Quantitative Data Synthesis

The strength of association between *XRCC1* Arg399Gln polymorphisms and HNSCC risk was measured by OR with 95% CI. Data were combined using both a fixed-effects model and a random-effects model [24]. Heterogeneity was assessed by the Cochrane *Q* statistics test. The fixed-effects model is applied when the effects are assumed to be homogenous, while the random-effects model is applied when they are heterogeneous. The potential publication bias was firstly estimated by visual inspection of the funnel plot. An asymmetric plot suggests a possible publication bias. The funnel plot asymmetry was assessed by the methods of Egger’s test and Begg’s test [25], [26]. We tested whether genotype frequencies of controls were in Hardy-Weinberg equilibrium (HWE) using the *χ^2^* test.

All of the statistical tests used in this meta-analysis were performed with Review Manager (Version 5.0.24.0, the Cochrane Collaboration) and STATA10.0 software package (Stata Corporation, College Station, Texas). All the tests were two-sided, a *P* value of less than 0.05 for any test was considered to be statistically significant.

## Results

### Meta-analysis Databases

A database was established according to the extracted information from all eligible articles. Essential information was listed in Table 1, which indicated the first author’s name, year of publication, source of control, country, ethnicity of subjects, the number of case and control and *P* value of HWE. There were a total of 18 studies with 3917 cases and 4560 controls concerning the *XRCC1* Arg399Gln polymorphisms related to HNSCC risk.

### Test of Heterogeneity

The heterogeneity of *XRCC1* codon 399 Gln/Gln vs. Arg/Arg, Arg/Gln vs. Arg/Arg and Gln/Gln+Arg/Gln vs. Arg/Arg was analyzed for 18 identified studies. The results showed that *XRCC1* codon 399 Gln/Gln vs. Arg/Arg, Arg/Gln vs. Arg/Arg and Gln/Gln+Arg/Gln vs. Arg/Arg for Caucasians and oral squamous cell carcinoma, Gln/Gln vs. Arg/Arg for healthy population-based control and larynx squamous cell carcinoma, and Arg/Gln vs. Arg/Arg for hospital-based control and healthy population-based control had no heterogeneity with a *P* value ≥0.05, therefore, we analyzed the summary odds ratios for them with a fixed-effects model. Random-effects model was used to analyze the summary ORs for the rest.

### Quantitative Data Synthesis

**Figure 1:**
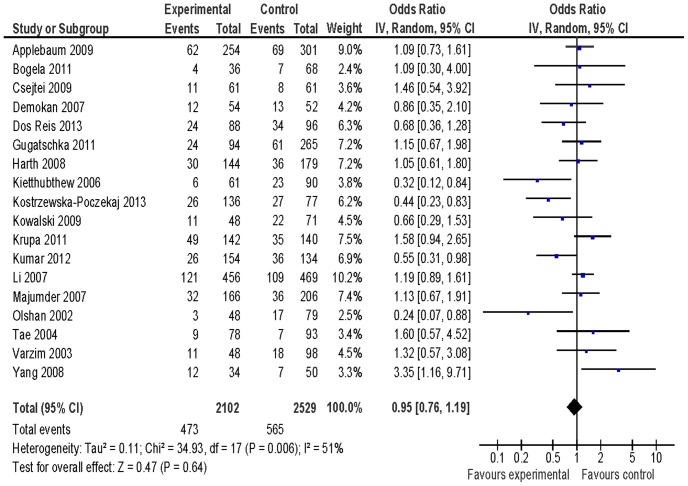
Forest plot of odds ratio for Gln/Gln vs. Arg/Arg of *XRCC1* Arg399Gln variants associated with HNSCC risk.

**Figure 2:**
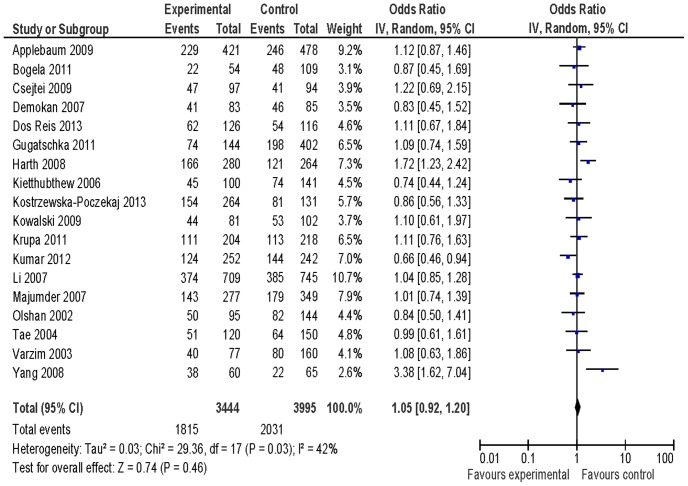
Forest plot of odds ratio for Arg/Gln vs. Arg/Arg of *XRCC1* Arg399Gln variants associated with HNSCC risk.

**Figure 3:**
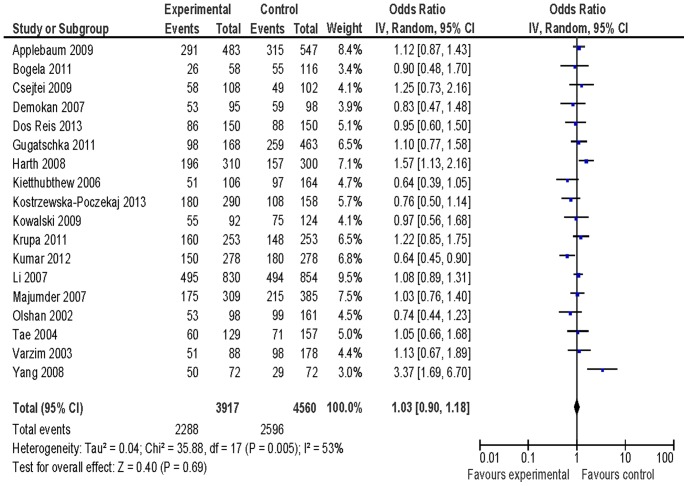
Forest plot of odds ratio for Gln/Gln+Arg/Gln vs. Arg/Arg of *XRCC1* Arg399Gln variants associated with HNSCC risk.

Table 2 listed the summary ORs of *XRCC1* Arg399Gln polymorphisms related to HNSCC risk on the basis of 3917 cases and 4560 controls. We did not observe any association of *XRCC1* Arg399Gln polymorphisms with HNSCC risk in the total population (OR = 0.95, 95% CI: 0.76–1.19 for Gln/Gln vs. Arg/Arg (Fig. 1), OR = 1.05, 95% CI: 0.92–1.20 for Arg/Gln vs. Arg/Arg (Fig. 2), and OR = 1.03, 95% CI: 0.90–1.18 for Gln/Gln+Arg/Gln vs. Arg/Arg (Fig. 3)) based on 18 identified studies including 3917 cases and 4560 controls. In subgroup analysis, we observed an increased risk of *XRCC1* codon 399 Arg/Gln genotype for HNSCC in Caucasians (OR = 1.20, 95% CI: 1.00–1.44) and Gln/Gln genotype for larynx squamous cell carcinoma (OR = 1.63, 95% CI: 1.10–2.40). We did not observe any association between *XRCC1* Arg399Gln variants and HNSCC risk in additional subgroup analyses (Table 2).

**Table 2:** Summery odds ratios on the relation of the *XRCC1* Arg399Gln polymorphisms to HNSCC risk.

Genotype		Case/Control	Heterogeneity test		Summery OR(95% CI)	Hypothesis test			Egger’s test		Begg’s test	
			*Chi^2^*	*P*		*Z* value	*P*	*df*	*t* value	*P*	*Z* value	*P*
Overall												
Gln/Gln vs. Arg/Arg		2102/2529	34.98	0.006	0.95(0.76–1.19)	0.47	0.64	17	1.00	0.330	0.45	0.649
Arg/Gln vs. Arg/Arg		3444/3995	29.36	0.03	1.05(0.92–1.20)	0.74	0.46	17	0.14	0.891	0.00	1.000
Gln/Gln+Arg/Gln vs. Arg/Arg		3917/4560	35.88	0.005	1.03(0.90–1.18)	0.40	0.69	17	0.22	0.829	0.38	0.705
Agreement with HWE												
Gln/Gln vs. Arg/Arg		2014/2433	33.46	0.006	0.97(0.77–1.22)	0.27	0.79	16	0.93	0.365	0.37	0.711
Arg/Gln vs. Arg/Arg		3318/3879	29.31	0.02	1.05(0.91–1.20)	0.67	0.50	16	0.11	0.916	0.04	0.967
Gln/Gln+Arg/Gln vs. Arg/Arg		3767/4410	35.72	0.003	1.03(0.89–1.19)	0.44	0.66	16	0.17	0.866	0.29	0.733
Gln/Gln vs. Arg/Arg		1199/1469	12.85	0.17	0.91(0.76–1.10)	0.99	0.32	9	0.20	0.847	0.36	0.721
Arg/Gln vs. Arg/Arg		2021/2321	16.71	0.05	1.06(0.94–1.20)	0.92	0.36	9	0.41	0.694	0.36	0.721
Gln/Gln+Arg/Gln vs. Arg/Arg		2279/2659	17.99	0.04	1.01(0.86–1.20)	0.17	0.87	9	0.51	0.625	0.36	0.721
Hospital												
Gln/Gln vs. Arg/Arg	903/1060		19.85	0.006	1.00(0.63–1.59)	0.01	0.99	7	0.80	0.455	0.12	0.902
Arg/Gln vs. Arg/Arg	1423/1674		12.63	0.08	1.06(0.85–1.32)	0.49	0.63	7	0.44	0.678	0.12	0.902
Gln/Gln+Arg/Gln vs. Arg/Arg	1638/1901		17.81	0.01	1.05(0.82–1.35)	0.40	0.69	7	0.07	0.947	0.12	0.902
Asian												
Gln/Gln vs. Arg/Arg	529/641		15.1	0.01	0.97(0.54–1.76)	0.09	0.93	5	0.59	0.582	0.60	0.548
Arg/Gln vs. Arg/Arg	863/1056		16.55	0.005	1.00(0.70–1.42)	0.01	0.99	5	1.07	0.334	0.60	0.548
Gln/Gln+Arg/Gln vs. Arg/Arg	952/1172		21.08	0.0008	1.00(0.68–1.46)	0.01	1.00	5	0.96	0.382	0.60	0.548
Caucasian												
Gln/Gln vs. Arg/Arg	531/751		8.23	0.14	0.91(0.69–1.20)	0.68	0.50	5	0.19	0.855	0.75	0.452
Arg/Gln vs. Arg/Arg	943/1153		7.05	0.22	1.20(1.00–1.44)	2.01	0.04	5	1.05	0.352	0.00	1.000
Gln/Gln+Arg/Gln vs. Arg/Arg	1056/1325		7.98	0.16	1.14(0.96–1.36)	1.53	0.13	5	0.85	0.444	0.38	0.707
Larynx												
Gln/Gln vs. Arg/Arg	260/356		2.38	0.50	1.63(1.10–2.40)	2.46	0.01	3	0.18	0.876	0.34	0.734
Arg/Gln vs. Arg/Arg	395/552		8.78	0.03	1.31(0.81–2.12)	1.10	0.27	3	0.79	0.511	0.34	0.734
Gln/Gln+Arg/Gln vs. Arg/Arg	471/619		9.07	0.03	1.38(0.87–2.19)	1.36	0.17	3	0.89	0.440	0.24	0.806
Oral												
Gln/Gln vs. Arg/Arg	369/444		5.41	0.14	0.78(0.55–1.09)	1.46	0.14	3	1.62	0.247	1.02	0.308
Arg/Gln vs. Arg/Arg	586/691		1.63	0.65	0.95(0.76–1.18)	0.48	0.63	3	0.85	0.483	0.34	0.734
Gln/Gln+Arg/Gln vs. Arg/Arg	660/797		2.75	0.43	0.90(0.73–1.11)	0.94	0.34	3	1.48	0.277	1.02	0.308

HWE: Hardy-Weinberg equilibrium.

### Bias Diagnosis

**Figure 4:**
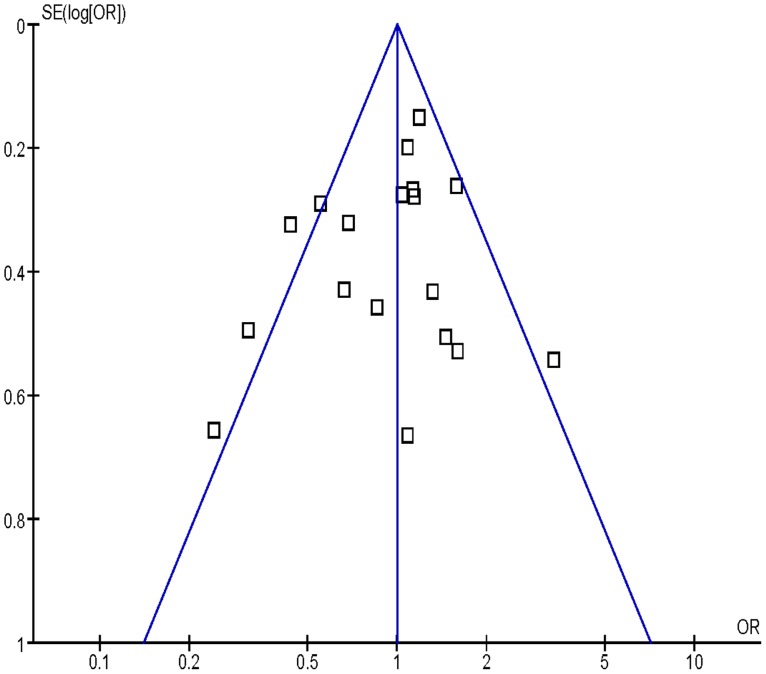
Funnel plot analysis to detect publication bias for Gln/Gln vs. Arg/Arg of *XRCC1* Arg399Gln variants associated with HNSCC risk.

**Figure 5:**
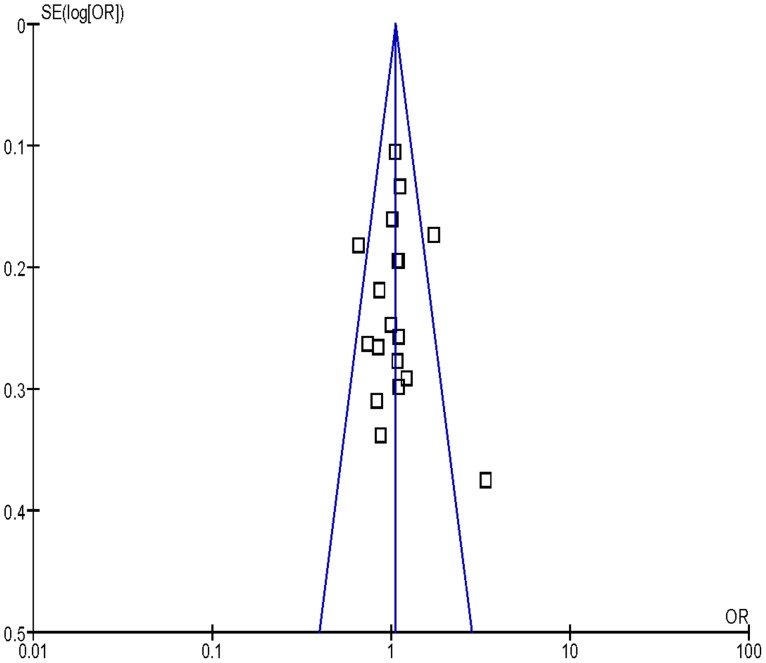
Funnel plot analysis to detect publication bias for Arg/Gln vs. Arg/Arg of *XRCC1* Arg399Gln variants associated with HNSCC risk.

**Figure 6:**
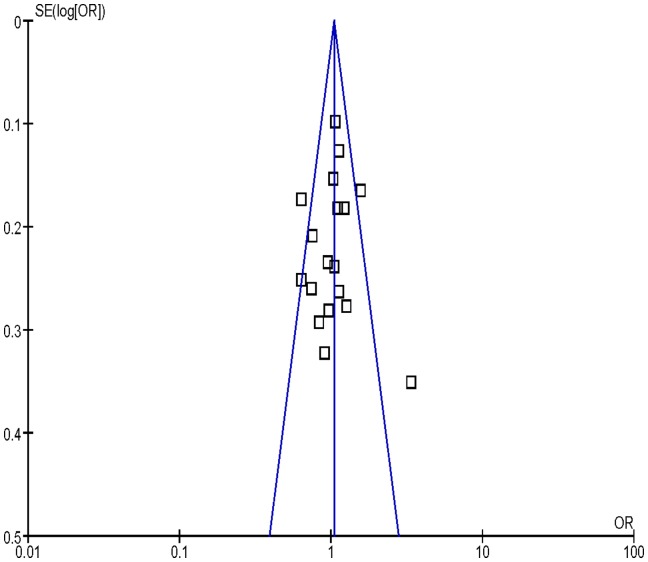
Funnel plot analysis to detect publication bias for Gln/Gln+Arg/Gln vs. Arg/Arg of *XRCC1* Arg399Gln variants associated with HNSCC risk.

Publication bias was assessed by funnel plot, the shape of the funnel plot seemed to be approximately symmetrical (Fig. 4, 5, 6). The results from Egger’s test and Begg’s test indicated that there were no obvious publication biases in our current meta-analysis (Table 2).

### Sensitivity Analysis

Sensitivity analyses were conducted to determine the influence of the individual dataset on the summary ORs by sequential removal of each eligible study. The overall effects were not altered when the studies were homogenous for Gln/Gln vs. Arg/Arg, Arg/Gln vs. Arg/Arg and Gln/Gln+Arg/Gln vs. Arg/Arg among total population by removing some eligible studies (Fig. S2, S3, S4).

## Discussion

XRCC1 is an important component of the base excision repair system, which is the predominant DNA repair pathway for small base lesions resulting from oxidation and alkylation damage [27]. The gene is located on chromosome 19q13.2–13.3, which has 32,354 base pairs and consists of 17 exons and 16 introns. It encodes a 70-kDa scaffolding protein consisting of 633 amino acids, which coordinates numerous protein-protein interactions, including with DNA ligase III and DNA polymerase at the site of damage [7], [28]. More than 300 validated single nucleotide polymorphisms in the XRCC1 gene were reported in the dbSNP database (http://www.ncbi.nlm.nih.gov/SNP), among them, rs25487 in *XRCC1* codon 399 of exon 10 was the most extensively studied polymorphic site. Recently, meta-analysis studies have reported that *XRCC1* Arg399Gln variant was associated with the risk of certain cancers, such as breast cancer [29], nasopharyngeal carcinoma [30], cervical carcinoma [31] and glioma [32]. In this paper, we performed a systematic literature review to comprehensively evaluate the association between sequence variants in *XRCC1* Arg399Gln and the risk of squamous cell carcinoma of the head and neck. We also estimated the possible effect modifications by source of control, ethnicity of subjects, tumor site and HWE in control. In summary, we did not observe an association of *XRCC1* Arg399Gln polymorphisms with HNSCC risk in the total population. Our findings are consistent with the previous meta-analysis study [5].

HNSCC includes tumors from different sites including oral, pharynx and larynx region, risk factors for these cancers may be different. For example, oral cavity and laryngeal cancers are mainly associated with tobacco use and alcohol consumption, while oropharyngeal cancers are principally related to viral infection, such as human papillomavirus (HPV) [33]. Unlike the HPV-negative oropharyngeal cancers, the HPV-positive subset is not related to tobacco or alcohol use, but with certain types of sexual behaviours. The HPV 16 subtype is present in up to 90% of HPV-related oropharyngeal cancers, while HPVs 18, 31 and 33 have been identified in the remainder. Recently, HPV has been recognized as a good prognostic factor in head and neck cancer, which has been attributed to several mechanisms, including absence of field cancerisation and increased sensitivity to chemoradiation therapy [34]. When subgroup analyses were conducted by tumor site, the subjects carrying Gln/Gln genotype had an increased risk in larynx squamous cell carcinoma subgroup. There was no association of *XRCC1* Arg399Gln polymorphisms with oral squamous cell carcinoma. We did not perform subgroup analyses on other tumor sites, owing to unavailable data.

Considering that the frequency of the *XRCC1* 399 Gln allele variant is significantly different among different ethnic population. Allele frequency patterns of *XRCC1* Arg399Gln polymorphism vary greatly among major ethnic groups. The frequency of Gln allele was more than 0.3 in Caucasians [35], [36], but less than 0.2 in Asian population [37], [38]. When stratified by ethnicity, we observed an association of *XRCC1* Arg399Gln polymorphisms with HNSCC risk among Caucasians, but not among Asian population. Different ethnicities cancer susceptibility associated with the *XRCC1* Arg399Gln polymorphisms was also observed in previous meta-analyses of lung cancer and breast cancer [35], [39]. This discrepancy among different ethnicities may be due to the different genetic backgrounds in these populations, subsequently leading to different genetic susceptibility to the same disease.

It is widely acknowledged that deviation from HWE may point to methodological weaknesses, such as biased selection of subjects or genotyping errors. The results of genetic association studies might be spurious if the distribution frequency of genotypes in the control groups was not in agreement with HWE [40]. To address this problem, subgroup analysis was conducted in this study by HWE in controls. When the study [13] that significantly deviated from HWE was excluded from this present analysis, we did not observe a substantial modification of the results, suggesting that this factor might not have effects on the overall estimates in the current meta-analysis.

This meta-analysis should be interpreted within the context of its limitations. First, only published articles were included in this study. Therefore, publication bias is very likely to occur. To address this issue, Egger’s test and Begg’s test were conducted. Our results indicated that the likelihood of key publication bias might not be present in this meta-analysis. Secondly, each study had different eligibility criteria for subjects and different source of controls, which should be taken into account while interpreting the combined estimates. When subgroup analysis was performed by source of control, we observed any association between *XRCC1* Arg399Gln polymorphisms and HNSCC risk neither in hospital-based control nor in healthy population-based control. Thirdly, the summary odds ratios were based on individual unadjusted estimates, while a more precise analysis might be performed if detailed individual data were available, which could allow for an adjusted estimation by sex, age, tobacco use, alcohol consumption and environment factors. In addition, the study number was limited and the sample size was relatively small in subgroup analyses, as a consequence, our estimates of the association in subgroup analyses might have occurred by chance. Thus, the results must be interpreted with caution.

In conclusion, this systematic review demonstrates that *XRCC1* Arg399Gln variants appear not to be a risk factor of HNSCC in the total population. However, *XRCC1* Arg399Gln variants might be a potential risk factor for HNSCC among Caucasians and for larynx squamous cell carcinoma. Large scale studies with the pooling of individual study data should be taken into account in the future studies to verify the results from this current meta-analysis.

## Supporting Information

Figure S1 **Flow diagram.**
(TIF)

Figure S2 **Sensitivity analysis for Gln/Gln vs. Arg/Arg.**
(TIF)

Figure S3 **Sensitivity analysis for Arg/Gln vs. Arg/Arg.**
(TIF)

Figure S4 **Sensitivity analysis for Gln/Gln+Arg/Gln vs. Arg/Arg.**
(TIF)

Checklist S1 **PRISMA 2009 Checklist.**
(DOC)
