# Supplementary figures and images for: Association of X-ray Repair Cross Complementing Group 1 Arg399Gln Polymorphisms with the Risk of Squamous Cell Carcinoma of the Head and Neck: Evidence from an Updated Meta-Analysis
Source: PLoS One. 2013 Oct 30;8(10):e77898. doi: 10.1371/journal.pone.0077898 (PMC3813759; doi:10.1371/journal.pone.0077898)

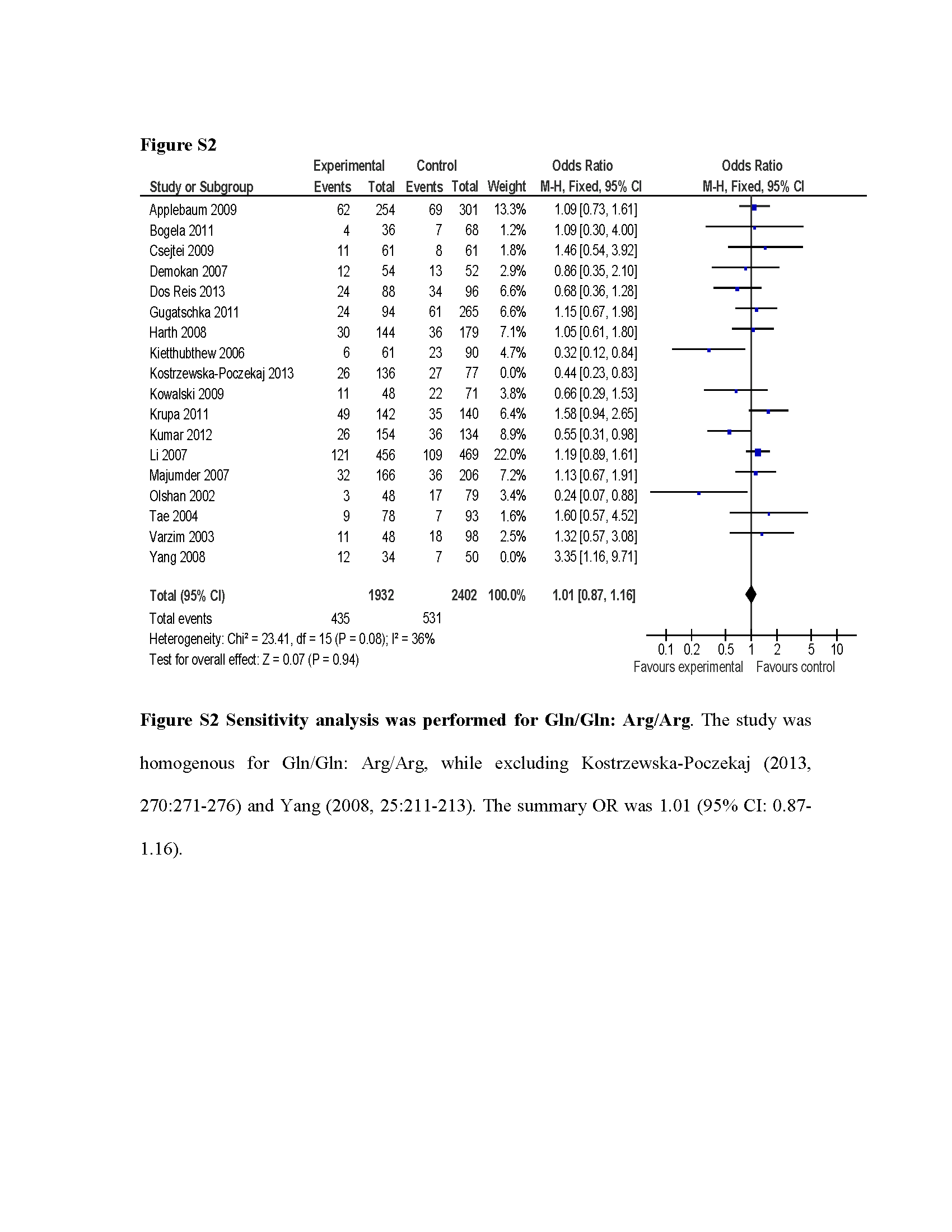

Supplement: Figure S2 — Sensitivity analysis for Gln/Gln vs. Arg/Arg. (TIF) [file pone.0077898.s002.tif]

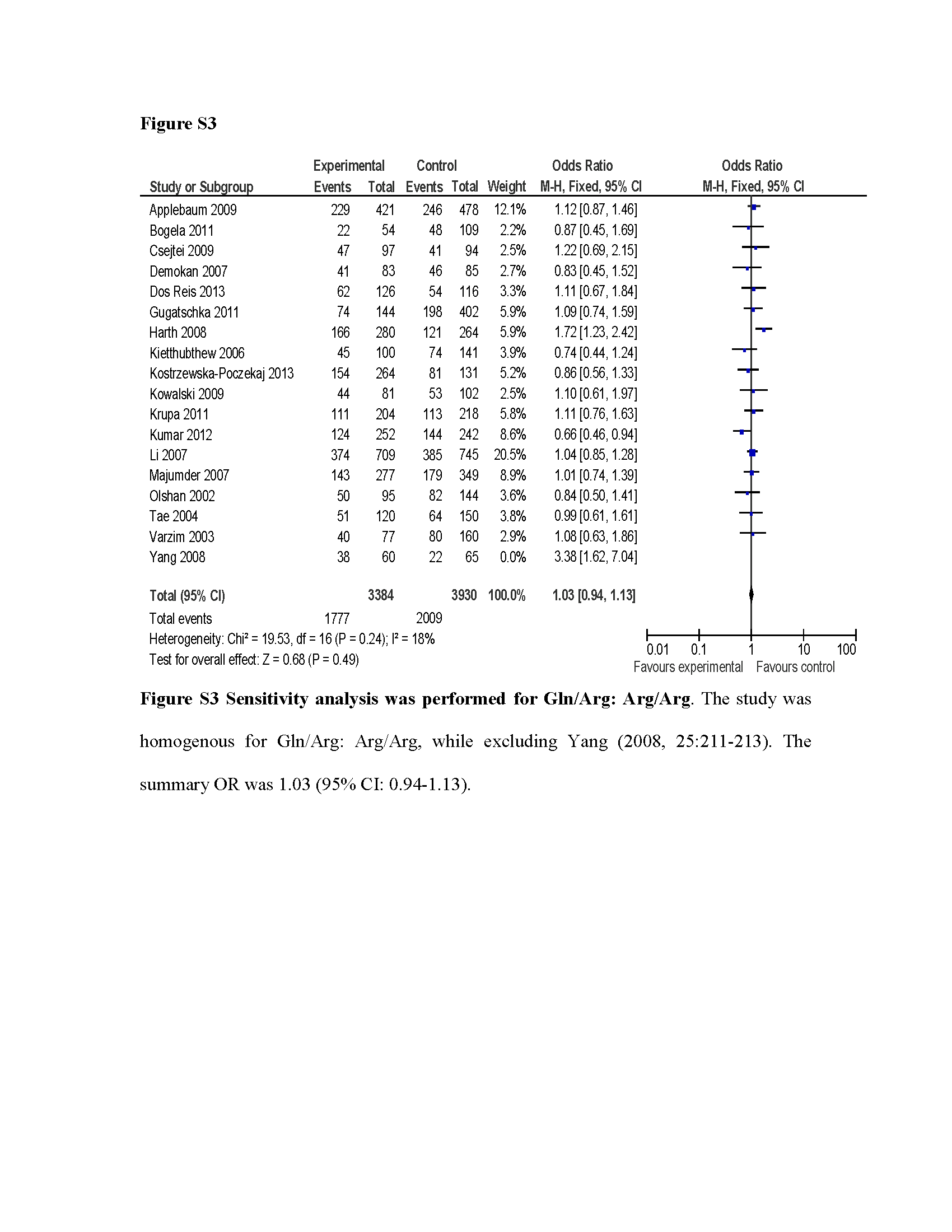

Supplement: Figure S3 — Sensitivity analysis for Arg/Gln vs. Arg/Arg. (TIF) [file pone.0077898.s003.tif]

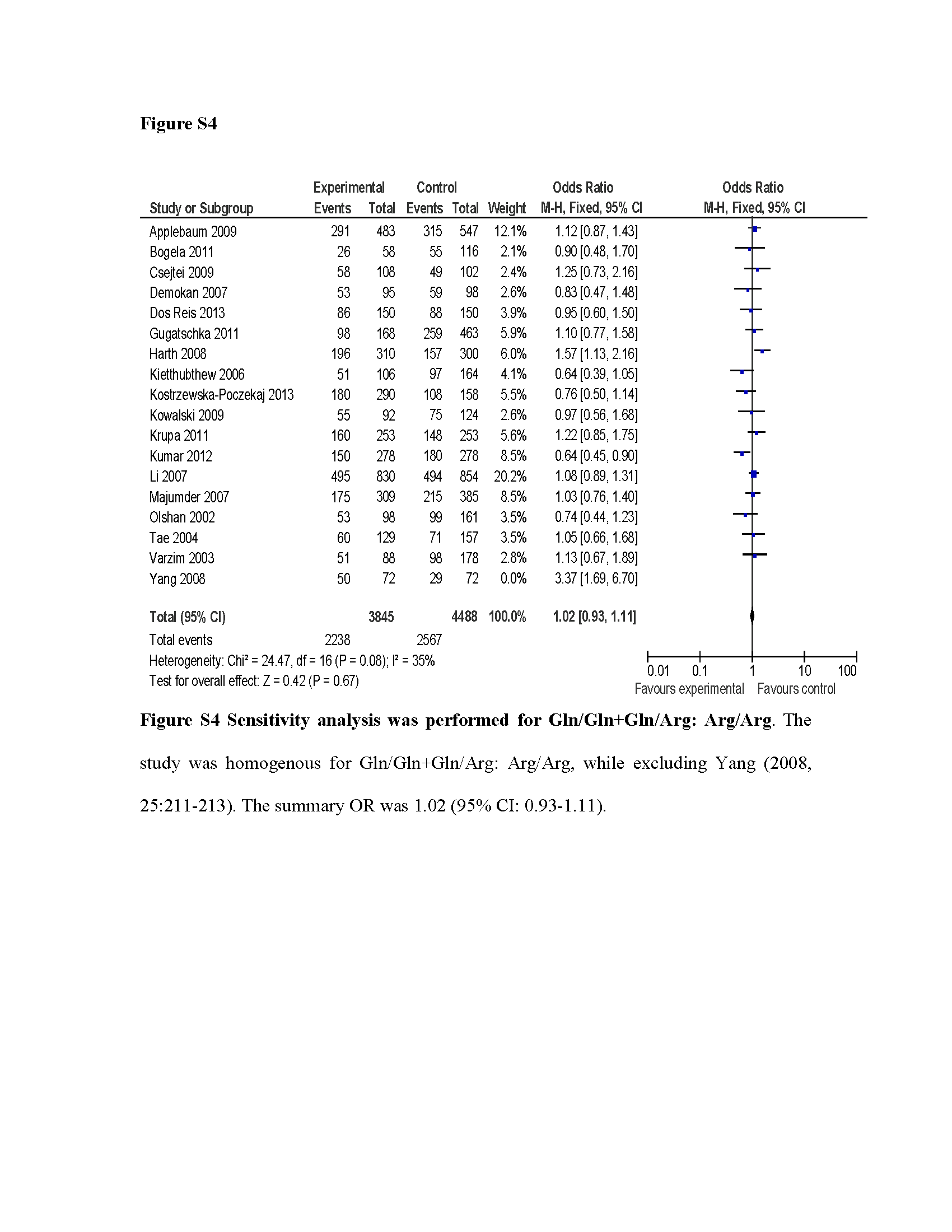

Supplement: Figure S4 — Sensitivity analysis for Gln/Gln+Arg/Gln vs. Arg/Arg. (TIF) [file pone.0077898.s004.tif]
